# Immunotherapy responsiveness and risk of relapse in Down syndrome regression disorder

**DOI:** 10.1038/s41398-023-02579-z

**Published:** 2023-08-08

**Authors:** Jonathan D. Santoro, Noemi A. Spinazzi, Robyn A. Filipink, Panteha Hayati-Rezvan, Ryan Kammeyer, Lina Patel, Elise A. Sannar, Luke Dwyer, Abhik K. Banerjee, Mellad Khoshnood, Saba Jafarpour, Natalie K. Boyd, Rebecca Partridge, Grace Y. Gombolay, Alison L. Christy, Diego Real de Asua, Maria del Carmen Ortega, Melanie A. Manning, Heather Van Mater, Gordan Worley, Cathy Franklin, Maria A. Stanley, Ruth Brown, George T. Capone, Eileen A. Quinn, Michael S. Rafii

**Affiliations:** 1 Division of Neurology, Children’s Hospital Los Angeles, Los Angeles, CA USA; 2 Department of Neurology, Keck School of Medicine of the University of Southern California, Los Angeles, CA USA; 3 Department of Pediatrics, Benioff Children’s Hospital, University of California San Francisco, San Francisco, CA USA; 4 Division of Child Neurology, UPMC Children’s Hospital of Pittsburgh, Pittsburgh, PA USA; 5 Division of Research on Children, Youth and Families, Keck School of Medicine of USC, Los Angeles, CA USA; 6 Department of Neurology, Children’s Hospital of Colorado, Aurora, CO USA; 7 Department of Psychiatry, University of Colorado School of Medicine, Aurora, CO USA; 8 Department of Psychiatry, University of Utah, Salt Lake City, UT USA; 9 Virginia Mason Health System, Issaquah, WA USA; 10 Department of Pediatrics, Division of Neurology Emory University and Children’s Healthcare of Atlanta, Atlanta, GA USA; 11 Providence Health System, Portland, OR USA; 12 Adult Down Syndrome Outpatient Clinic, Department of Internal Medicine, Fundación de Investigación Biomédica, Hospital Universitario de La Princesa, Madrid, Spain; 13 Department of Psychiatry, Clinica Universidad de Navarra, Madrid, Spain; 14 Department of Pediatrics, Stanford University School of Medicine, Palo Alto, CA USA; 15 Division of Rheumatology, Department of Pediatrics, Duke University School of Medicine, Durham, NC USA; 16 Division of Pediatric Neurology and Developmental Medicine, Department of Pediatrics, Duke University School of Medicine, Durham, NC USA; 17 Queensland Center for Intellectual and Developmental Disability, Mater Research Institute, The University of Queensland, South Brisbane, QLD Australia; 18 Department of Pediatrics, University of Wisconsin School of Medicine and Public Health, Madison, WI USA; 19 Department of Psychology, Virginia Commonwealth University, Richmond, VA USA; 20 Department of Pediatrics, Johns Hopkins School of Medicine, Baltimore, MD USA; 21 Department of Pediatrics, University of Toledo College of Medicine and Life Science, Toledo, OH USA; 22 Alzheimer’s Therapeutic Research Institute (ATRI), Keck School of Medicine at the University of Southern California, San Diego, CA USA

**Keywords:** Psychiatric disorders, Clinical genetics

## Abstract

Down syndrome regression disorder (DSRD) is a clinical symptom cluster consisting of neuropsychiatric regression without an identifiable cause. This study evaluated the clinical effectiveness of IVIg and evaluated clinical characteristics associated with relapse after therapy discontinuation. A prospective, multi-center, non-randomized, observational study was performed. Patients met criteria for DSRD and were treated with IVIg. All patients underwent a standardized wean-off therapy after 9–12 months of treatment. Baseline, on-therapy, and relapse scores of the Neuropsychiatric Inventory Total Score (NPITS), Clinical Global Impression-Severity (CGI-S), and the Bush–Francis Catatonia Rating Scale (BFCRS) were used to track clinical symptoms. Eighty-two individuals were enrolled in this study. Patients had lower BFCRS (MD: −6.68; 95% CI: −8.23, −5.14), CGI-S (MD: −1.27; 95% CI: −1.73, −0.81), and NPITS scores (MD: −6.50; 95% CI: −7.53, −5.47) while they were on therapy compared to baseline. Approximately 46% of the patients (*n* = 38) experienced neurologic relapse with wean of IVIg. Patients with neurologic relapse were more likely to have any abnormal neurodiagnostic study (*χ*^2^ = 11.82, *P* = 0.001), abnormal MRI (*χ*^2^ = 7.78, *P* = 0.005), and abnormal LP (*χ*^2^ = 5.45, *P* = 0.02), and a personal history of autoimmunity (OR: 6.11, *P* < 0.001) compared to patients without relapse. IVIg was highly effective in the treatment of DSRD. Individuals with a history of personal autoimmunity or neurodiagnostic abnormalities were more likely to relapse following weaning of immunotherapy, indicating the potential for, a chronic autoimmune etiology in some cases of DSRD.

## Introduction

Down syndrome (DS) is the most common cause of intellectual disability worldwide and occurs in 1 in 800 live births in the United States [1]. Neurologic and psychiatric diseases in this population are well described, although the last decade has seen an increasing frequency of reports of the onset of subacute developmental regression of unclear etiology in individuals considered too young to develop Alzheimer’s disease and too old to develop autism spectrum disorder. This condition has been referred to as Down Syndrome Regression Disorder (DSRD) and has primarily been reported in young persons with DS between ages 10 and 30 years [2, 3]. Symptoms include a subacute loss of previously acquired developmental skills in the areas of language, communication, cognition, executive function, behavioral, and adaptive skills [2, 4–7]. Other symptoms can include psychiatric manifestations, bradykinesia, catatonia, and rapid-onset insomnia [4, 5, 7, 8]. DSRD can be severe and significantly impact both the quality of life and autonomy of persons with DS.

Therapeutic interventions for this condition are broad and have ranged from antipsychotics to immunotherapy [4, 9]. A minority of individuals with DSRD may have a neuroinflammatory etiology to the disease, confirmed by the presence of abnormal neurodiagnostic studies and dramatic immunotherapy responsiveness in some patients [4, 9, 10]. Prior studies have identified that up to 40% of individuals with DSRD have neuroimaging (e.g., T2 signal prolongation or GRE signal abnormalities in the basal ganglia) or cerebrospinal fluid (e.g., pleocytosis, oligoclonal banding, high IgG index, etc.) abnormalities and that when these are present, individuals are more than four times more likely to respond to immunotherapy than other patients [4]. Identification of these findings, which are indicative of neuroinflammation, were a primary rationale for initial studies using immunotherapy in individuals with symptoms of DSRD [4, 10]. While immunotherapy provides a tool to rapidly reverse this clinical syndrome, guidance on dosing and duration of therapy remains unclear.

This study sought to examine changes in clinical measures of functionality, gait, catatonia, and neuropsychiatric symptoms among individuals with DSRD receiving IVIg, investigate possible demographic, lab, and clinical factors linked to responsiveness to immunotherapy with IVIg, and assess the likelihood of successful treatment tapering once improvement of symptoms has been achieved.

## Materials and methods

### IRB and data availability

IRB approval was obtained for this study with waived assent authorized in patients not capable of providing assent. Consent was obtained from caregivers (if <18 years) or legal guardians (if >18 years) when assent could not be obtained. Anonymized data are available to qualified researchers upon request.

### Participants and study design

All individuals evaluated in the DS neurology clinic at multiple institutions were evaluated for participation in this study. Inclusion criteria included age between 8 and 26 years at the time of symptom onset, diagnosis of either possible or probable DSRD per expert consensus guidelines [3], and completion of clinical neurodiagnostic studies (EEG, MRI, and Lumbar Puncture (LP)). Confirmation of diagnosis was performed by an arbiter with no knowledge of the case (MK). Exclusion criteria which included, age <8 or >26 years at the time of symptom onset, active cardiac or pulmonary disease, frequent infection (defined as more than two infections requiring antibiotics or antivirals per year), a history of neoplasia or receipt of chemotherapy, structural brain malformation on neuroimaging, active or a history of epilepsy (excluding febrile seizure), current use of electroconvulsive therapy and use of any immunotherapy not related to DSRD. Previously published cases of individuals receiving immunotherapy were also excluded [4, 9]. Patients were permitted to be on psychotropic medications (e.g., benzodiazepines, selective serotonin reuptake inhibitor, antipsychotics, etc.), although once started on immunotherapy, dosing was locked with the exception of weaning if indicated. Individuals with co-morbid diagnoses of ASD were not excluded, although they were all required meeting consensus criteria for DSRD.

Demographic data, medical history, and results of clinical and diagnostic investigations were collected through clinical documentation. Radiographic data was reviewed independently by a board-certified neuroradiologist. This study did not involve a control population of children with DS without DSRD as there are no other established indications for IVIg in DS.

### Visit schedule

Prior to enrollment in the study, all patients were clinically evaluated and diagnosed with DSRD as per published guidelines (baseline) [3]. Patients were evaluated clinically at +0 days (baseline), +90 days, and +180 days (+/− 7 days) after the initiation of IVIg. In addition to these scheduled visits, patients had the option for more frequent urgent visits when necessary. Behavioral and neuropsychiatric testing assessments were performed at baseline and at +180 days. During the titration period, patients were evaluated at standardized time points of +35 days, +77 days, and +119 days at the time of subsequent infusions (+/− 7 days). Behavioral and neuropsychiatric assessments were performed at the time of clinical relapse (urgent evaluation) or +119 days, whichever came first. A representation of the evaluation and therapeutic interventions is presented in Appendix A.

### Behavioral/neuropsychiatric assessment

Given that DSRD has a wide variety of presenting symptoms, we employed several validated study tools in tandem to capture differences in disease severity. Research coordinators administered the Clinical Global Impression-Severity (CGI-S) scale at all clinical visits. At the baseline visit, the severity scale was performed and during follow-up visits the improvement scale versions of this 7-point scale were administered. This scale is a self-reported global assessment of disease activity and provided a validated objective assessment of clinical improvements over time as well as parental impression of disease severity. This metric has been used in other studies evaluating efficacy of interventions in nonverbal or intellectually disabled populations [11]. Physician evaluators also completed the Neuropsychiatric Inventory Questionnaire (NPI-Q) and the Bush–Francis Catatonia Rating Scale (BFCRS) at all clinical visits. The NPI-Q is a well-validated measure of multiple domains of neuropsychiatric disturbance including fields of delusions, hallucinations, agitation, depression, anxiety, elation, apathy, disinhibition, motor abnormalities, nocturnal behaviors, and eating behaviors. The broad range of assessment domains and prior utilization in persons with DS made this an ideal endpoint [12, 13]. The BFCRS is a well-established and validated tool for assessment of catatonia which is present in up to 70% of individuals with DSRD [4, 9]. The BFCRS is a 23-point physical examination administered by physicians evaluating multiple symptoms of catatonia which has also been used in early cohorts of children with symptoms of DSRD, making it an ideal longitudinal measure [14, 15]. To assess global motor impairment, patients also had a timed 25-foot walk (25FTW) completed as part of their clinical evaluations when able to participate and follow directions. Higher scores for each of these metrics (CGI-S, NPI-Q, BFCRS, and 25FTW) was indicative of increased severity of symptoms.

### Definitions of abnormal neurodiagnostics

#### Electroencephalogram (EEG)

Focal or generalized slowing, focal epileptiform discharges out of any cortex, or seizures were considered abnormal. Generalized discharges were considered abnormal, although inconsistent with the diagnosis of DSRD. All patients had to have at least one prior EEG that did not demonstrate these results previously.

#### MRI

All MRIs had to be performed on a 3T scanner with and without contrast administration. Any abnormality beyond a structural malformation (e.g., Chiari malformation) was considered abnormal. Patients did not require a prior “normal” MRI.

#### Lumbar puncture (LP)

Abnormalities were defined as having any of the following: WBC count >5 cells/mm^3^, total protein >60 mg/dL, presence of oligoclonal bands, an IgG index of >0.66, and/or an elevated neopterin (>33 nmol/mL). Samples with over 1000 RBCs were excluded from the analysis. Patients did not require a prior “normal” LP.

### Therapeutic interventions

A high-concentration formulation of IVIg was utilized in all patients (10%, 100 mg/dL) at each dosing period. Patients were administered either Gammagard, Privagen, or Octagam formulations of IVIg, depending on local infusion policies and regional restrictions on use. Once a patient was started on a particular formulation of IVIg, they had to continue on that same formulation unless an infusion reaction occurred. IVIg was dosed at 2 g/kg (administered over 2 days) for the induction dose followed by 1 g/kg (administered over one day) for maintenance dosing as per prior dosing regimens in pediatric inflammatory neurologic disease [16, 17]. The timeframe between maintenance doses was 28 days +/−3 days and infusion protocols are presented in Appendix B. Steroids were not co-administered for any infusion unless as a treatment for medication reaction. Infusions could be administered at an outpatient infusion center or at home. In all situations, infusions were administered by a registered nurse.

### Therapeutic wean

All patients were weaned off IVIg using a standard protocol. After 9 months of IVIg therapy, the frequency of infusions was reduced from every 4 to every 5 weeks, then to every 6 weeks, then to every 7. Completed wean-off therapy would take 18 total weeks. If there was no clinical return of symptoms (e.g., catatonia, mutism etc.), IVIg therapy was then discontinued; if there was recurrence of symptoms, the patient was placed back on an every 4-week infusion schedule. A relapse was defined as any sustained worsening (≥3 days) in any of the symptoms listed on the international DSRD criteria checklist and was determined by the evaluating clinician [3].

### Safety assessments

Patients were asked standardized screening questions to report any adverse events (AE) on therapy at two time points ( + 90 days and +180 days (+/− 7 days)) during the study period; they were also asked about intercurrent use of antibiotics or antivirals, urgent or emergent medical care evaluations, hospitalizations, or febrile illnesses. All potential AEs were reported.

During infusions, a nurse was available at bedside for rapid triage of reactions to IVIg administration. All infusion reactions were evaluated and escalated to the treating physician when appropriate. At the first sign of an infusion reaction, the infusion was paused, and only resumed after medical clearance by the supervising physician.

### Statistical analysis

The primary outcomes of interest were the 25FTW, BFCRS, CGI-S, NPI-Q Total Scores (NPITS) collected at multiple time points (i.e., baseline, on therapy, and after therapy). These scores were analyzed using mixed-effects regression models with an unstructured covariance model for the “within-subject repeated measures” and “indicators for time post-baseline to capture change in outcome mean scores from baseline”. The models further allowed for fixed effects for demographics and disease biometrics and their interactions with time, with effects added individually.

Additional mixed-effects models with similar covariance structure were used including the fixed effects for those with relapse relative to those without, categorical indicators for time post-baseline, and the interaction between relapse and time. The models further allowed for interaction effects between demographics and disease biometrics and relapse and time.

Demographics, disease biometrics, and baseline clinical features were compared between patients with and without neurologic relapse using chi-square (*χ*^2^) or Fisher’s exact tests for categorical variables, and *t* test for continuous variables. Univariate logistic regression was used to model the association between relapse and individual demographics, disease biometrics, and baseline clinical features. Factors that differed significantly by relapse were entered into a separate multivariable logistic regression model. All analyses were conducted using Stata/MP version-17.0 (StataCorp. 2021. College Station, TX, StataCorp LLC.).

## Results

### Demographics and clinical features

Ninety-three patients were identified for potential review, of which 82 (88%) met all inclusion criteria. The most common reasons for exclusions were age >26 years at symptom onset (*n* = 8, 73%), prior receipt of immunotherapy unrelated to DSRD (*n* = 2, 18%), and history of epilepsy (*n* = 1, 9%). Four patients included were treated with immunotherapy previously for their DSRD symptoms, although all four received intermittent or incomplete immunotherapy plans (e.g., single administration of IVIg) at least 6 months prior to the start of this study. Demographics and clinical features of cases are reported in Table 1.

**Table 1:** Demographics and clinical data (*N* = 82).

	No relapse (*n* = 44; 53.7%)		Relapse (*n* = 38; 46.3%)		All (*n* = 82)	
*Demographics*						
Sex						
Female	26	59.1%	18	47.4%	44	53.7%
Male	18	40.9%	20	52.6%	38	46.3%
Race						
Caucasian	34	77.3%	31	81.6%	65	79.3%
Black	6	13.6%	4	10.5%	10	12.2%
Asian	4	9.1%	3	7.9%	7	8.5%
Ethnicity						
Non-Hispanic	13	29.5%	13	34.2%	26	31.7%
Hispanic	31	70.5%	25	65.8%	56	68.3%
Age at symptom onset	14.1	(3.5)	15.6	(4.6)	14.8	(4.1)
Age at diagnosis	16.5	(4.1)	17.9	(5.1)	17.2	(4.6)
Age at therapy	16.8	(4.1)	18.4	(4.9)	17.5	(4.5)
Δ Age at symptom onset and age at diagnosis	2.4	(1.9)	2.3	(1.8)	2.4	(1.8)
Δ Age at therapy and age at diagnosis^*^	*0.3*	*(0.5)*	*0.5*	*(0.6)*	*0.4*	*(0.6)*
Δ Age at therapy and age at symptom onset	2.7	(2.1)	2.8	(1.7)	2.8	(1.9)
Time (months) to symptom peak	3.7	(2.5)	3.3	(2.5)	3.5	(2.5)
Trigger present	20	45.5%	20	52.6%	40	48.8%
Type of trigger						
Infection	9	20.5%	7	18.4%	16	19.5%
Change of school/work/home environment	6	13.6%	5	13.2%	11	13.4%
Loss of family/caregiver/friend	2	4.5%	2	5.3%	4	4.9%
Death	1	2.3%	2	5.3%	3	3.7%
Change in residence	0	0.0%	2	5.3%	2	2.4%
Abuse	1	2.3%	1	2.6%	2	2.4%
Medical change	1	2.3%	1	2.6%	2	2.4%
*Disease biometrics*						
Probable DSRD criteria	17	38.6%	13	34.2%	30	36.6%
History of personal autoimmune disease	18	45.0%	15	46.9%	33	45.8%
Serum cytokines	7	26.9%	13	54.2%	20	40.0%
Catatonia	30	68.2%	30	78.9%	60	73.2%
EEG abnormal^*^	*7*	*17.5%*	*12*	*37.5%*	*19*	*26.4%*
MRI abnormal^**^	**4**	**10.0%**	**12**	**37.5%**	**16**	**22.2%**
Lumbar puncture abnormal^**^	**3**	**7.5%**	**9**	**28.1%**	**12**	**16.7%**
Any neurodiagnostic study abnormal^**^	**9**	**22.5%**	**20**	**62.5%**	**29**	**40.3%**
Psychotropic medications at baseline	35	79.5%	30	78.9%	65	79.3%
Benzodiazepines	22	50%	17	44.7%	39	47.6%
SSRI/SNRIs	17	38.6%	10	26.3%	27	32.9%
Antipsychotics	8	18.2%	7	18.4%	17	20.7%
Anticonvulsants	2	4.5%	5	13.1%	7	8.5%
Mood stabilizers	3	6.8%	0	0%	3	3.6%
Prior immunotherapy	3	6.8%	1	2.6%	4	4.9%
IVIg brand						
Gammaguard	27	61.4%	28	73.7%	55	67.1%
Octagam	11	25.0%	7	18.4%	18	22.0%
Privagen	6	13.6%	3	7.9%	9	11.0%
IVIg duration	7.5	(2.4)	7.8	(2.1)	7.6	(2.3)
*Baseline clinical features*						
25-Foot walk^**^	**9.4**	**(4.5)**	**12.3**	**(6.4)**	**10.7**	**(5.6)**
Bush–Francis Severity Score	16.6	(9.9)	19.5	(10.9)	17.9	(10.4)
CGI severity of illness^**^	**3.3**	**(1.3)**	**4.1**	**(1.5)**	**3.6**	**(1.4)**
NPI Total Score^**^	**18.7**	**(5.7)**	**21.8**	**(5.9)**	**20.1**	**(6.0)**
NPI delusions	3.8	(1.9)	3.6	(1.6)	3.7	(1.8)
NPI hallucinations^*^	*1.2*	*(1.4)*	*1.8*	*(1.7)*	*1.5*	*(1.5)*
NPI agitation	2.1	(1.4)	1.9	(1.6)	2.0	(1.5)
NPI anxiety	0.8	(0.9)	0.6	(0.7)	0.7	(0.8)
NPI apathy^*^	*2.5*	*(1.5)*	*3.1*	*(1.6)*	*2.8*	*(1.5)*
NPI irritability^**^	**1.7**	**(1.2)**	**2.5**	**(1.5)**	**2.1**	**(1.4)**
NPI euphoria	0.5	(0.8)	0.6	(0.8)	0.5	(0.8)
NPI disinhibition	0.5	(0.9)	0.7	(1.4)	0.5	(1.2)
NPI aberrant motor	2.6	(1.7)	3.3	(1.9)	2.9	(1.8)
NPI night time	2.3	(1.7)	2.7	(1.5)	2.5	(1.6)
NPI appetite/eating	0.7	(1.0)	1.0	(1.5)	0.9	(1.2)

*P < 0.1 (italic font); **P < 0.05 (bold font). Data are mean (SD) or frequency and percentage %. The frequency and percentages of incomplete variables are as follows: history of personal autoimmune disease (*n* = 10; 12.2%), serum cytokines (*n* = 32; 39%), EEG abnormal (*n* = 10; 12.2%), MRI abnormal (*n* = 10; 12.2%), lumbar puncture abnormal (*n* = 10; 12.2%), and any neurodiagnostic study abnormal (*n* = 10; 12.2%). Δ = difference between. Multiple responses allotted for types of trigger and psychotropic medications at baseline.

### Therapeutics and safety

Gammagard was the most common IVIg formulation administered (67%). In total, only two patients (2.4%) had AEs reported during the study period. One patient had an infusion reaction (rash) during the third infusion and one developed wheezing two hours into her fourth infusion. Infusions were temporarily paused but completed after therapeutic intervention. Both patients continued to receive infusions with no further AEs. In total, there were six occasions where patients were co-administered steroids during an infusion as a reaction medication out of 738 (0.5%). Reasons for administration were pruritis (4/6, 67%), rash (1/6, 17%), and wheezing (1/6, 17%). No participant developed deep venous thrombosis or clotting, headache, aseptic meningitis, or other known side effects of IVIg. Nearly 20% (*n* = 16) of patients’ caregivers reported subjective improvements in skin conditions such as hidradenitis suppurativa, eczema, and psoriasis. This was not systematically asked by clinicians but was information volunteered by families in some circumstances.

### Therapeutic effects

**Fig. 1:**
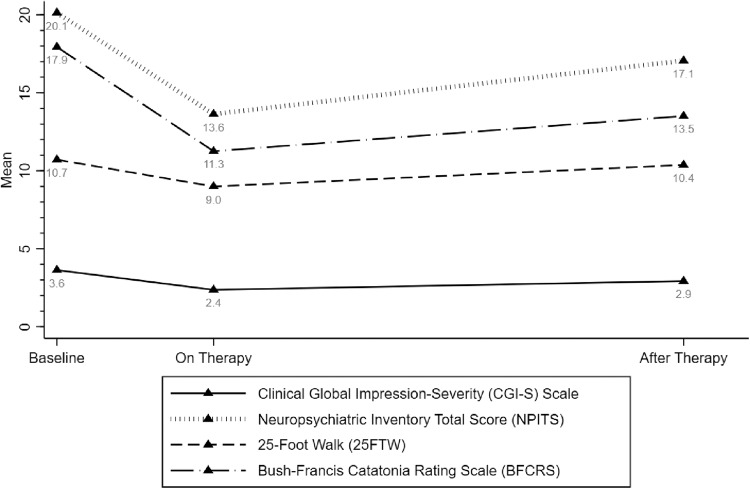
Therapeutic response to immunotherapy. Clinical features, including behavioral and neuropsychiatric assessments over the study period.

Changes in primary clinical outcome measures are displayed in Fig. 1 and Table 2. While on therapy, in comparison to baseline, patients had lower scores for 25FTW (mean difference (MD): −1.72; 95% confidence interval (CI): −2.42, −1.01), BFCRS (MD: −6.68; 95% CI: −8.23, −5.14), CGI-S (MD: −1.27; 95% CI: −1.73, −0.81), and NPITS (MD: −6.50; 95% CI: −7.53, −5.47). Furthermore, after therapy, lower mean scores were observed for BFCRS (MD: −4.43; 95% CI: −5.89, −2.97), CGI-S (MD: −0.71; 95% CI: −0.95, −0.47), and NPITS (MD: −3.07; 95% CI: −3.91, −2.23) but not 25FTW (MD: −0.34; 95% CI: −0.91, 0.24), compared to baseline.

**Table 2:** Estimated regression coefficient with 95% confidence interval (CI) from repeated measures analyses for all patients.

	Coef.	SE	95% CI	*P* value
25-Foot walk				
Prior to Therapy	0.00	0.00		
On Therapy^**^	**−1.72**	**0.36**	**[−2.42, −1.01]**	**0.0000**
After Therapy	−0.34	0.29	[−0.91, 0.24]	0.2509
Bush–Francis Score				
Prior to Therapy	0.00	0.00		
On Therapy^**^	**−6.68**	**0.79**	**[−8.23, −5.14]**	**0.0000**
After Therapy^**^	**−4.43**	**0.75**	**[−5.89, −2.97]**	**0.0000**
CGI-Severity Score				
Prior to Therapy	0.00	0.00		
On Therapy^**^	**−1.27**	**0.23**	**[−1.73, −0.81]**	**0.0000**
After Therapy^**^	**−0.71**	**0.12**	**[−0.95, −0.47]**	**0.0000**
Total NPI Score				
Prior to Therapy	0.00	0.00		
On Therapy^**^	**−6.50**	**0.53**	**[−7.53, −5.47]**	**0.0000**
After Therapy^**^	**−3.07**	**0.43**	**[−3.91, −2.23]**	**0.0000**

**P < 0.05 (bold font). The repeated measure analyses were carried out using longitudinal mixed-effects regression models with restricted maximum likelihood estimation and Kenward–Roger method for small-sample adjustment.

### Clinical response variables

There was evidence that changes in clinical responses differed by disease-related factors, such as the presence of catatonia and treatment with prior immunotherapy as well as neurodiagnostic study abnormalities. Clinical responses were more profound in individuals with catatonia, those who had received prior immunotherapy for the treatment of DSRD, and those with any neurodiagnostic study abnormality (Table 3).

**Table 3:** Estimated means with 95% confidence interval (CI) for clinical outcomes differed by potential disease biometrics.

	On therapy				After therapy			
	Mean change from baseline	SE	95% CI	*P* value	Mean change from baseline	SE	95% CI	*P* value
25-Foot walk								
With catatonia	**−2.39**	**0.40**	**[−3.17, −1.62]**	**0.0000**	**−0.75**	**0.33**	**[−1.40, −0.09]**	**0.0254**
Without catatonia	0.13	0.65	[−1.15, 1.41]	0.8457	0.78	0.55	[−0.30, 1.86]	0.1580
With any neurodiagnostic abnormalities	**−3.14**	**0.60**	**[−4.32, −1.96]**	**0.0000**	0.31	0.551	[−0.68, 1.30]	0.5397
Without any neurodiagnostic abnormalities	**−1.00**	**0.50**	**[−1.97, −0.02]**	**0.0447**	**−0.86**	**0.42**	**[−1.68, −0.05]**	**0.0379**
Prior immunotherapy	**−4.05**	**1.61**	**[−7.21, −0.89]**	**0.0120**	**−4.07**	**1.26**	**[−6.55, −1.60]**	**0.0013**
No prior immunotherapy	**−1.60**	**0.37**	**[−2.31, −0.88]**	**0.0000**	−0.14	0.29	[−0.71, 0.42]	0.6130
Bush–Francis Score								
With catatonia	**−8.45**	**0.84**	**[−10.10, −6.80]**	**0.0000**	**−5.45**	**0.85**	**[−7.11, −3.79]**	**0.0000**
Without catatonia	−1.86	1.39	[−4.59, 0.87]	0.1811	−1.64	1.40	[−4.38, 1.11]	0.2432
With any neurodiagnostic abnormalities	**−9.86**	**1.30**	**[−12.41, −7.32]**	**0.0000**	**−3.45**	**1.29**	**[−5.98, −0.92]**	**0.0075**
Without any neurodiagnostic abnormalities	**−5.28**	**1.07**	**[−7.37, −3.19]**	**0.0000**	**−5.56**	**1.06**	**[−7.63, −3.48]**	**0.0000**
EEG abnormal	**−10.42**	**1.62**	**[−13.60, −7.24]**	**0.0000**	**−6.00**	**1.60**	**[−9.14, −2.86]**	**0.0002**
EEG normal	**−5.94**	**0.97**	**[−7.85, −4.04]**	**0.0000**	**−4.25**	**0.96**	**[−6.12, −2.37]**	**0.0000**
MRI abnormal	**−8.44**	**1.83**	**[−12.02, −4.85]**	**0.0000**	−1.56	1.70	[−4.90, 1.78]	0.3590
MRI normal	**−6.75**	**0.98**	**[−8.67, −4.83]**	**0.0000**	**−5.61**	**0.91**	**[−7.39, −3.82]**	**0.0000**
LP abnormal	**−9.42**	**1.41**	**[−12.17, −6.66]**	**0.0000**	−1.67	1.15	[−3.92, 0.59]	0.1470
LP normal	**−6.25**	**0.63**	**[−7.48, −5.02]**	**0.0000**	**−3.55**	**0.51**	**[−4.56, −2.54]**	**0.0000**
Prior immunotherapy	**−9.00**	**3.58**	**[−16.01, −1.99]**	**0.0119**	**−11.25**	**3.31**	**[−17.73, −4.77]**	**0.0007**
No prior immunotherapy	**−6.56**	**0.81**	**[−8.15, −4.98]**	**0.0000**	**−4.08**	**0.75**	**[−5.54, −2.61]**	**0.0000**
CGI-Severity Score								
With catatonia	**−1.58**	**0.27**	**[−2.10, −1.06]**	**0.0000**	**−0.63**	**0.14**	**[−0.92, −0.35]**	**0.0000**
Without catatonia	−0.41	0.44	[−1.27, 0.45]	0.3519	**−0.91**	**0.24**	**[−1.38, −0.44]**	**0.0001**
With any neurodiagnostic abnormalities	**−2.21**	**0.38**	**[−2.96, −1.46]**	**0.0000**	**−0.45**	**0.21**	**[−0.86, −0.04]**	**0.0324**
Without any neurodiagnostic abnormalities	**−0.81**	**0.31**	**[−1.43, −0.20]**	**0.0096**	**−1.00**	**0.17**	**[−1.34, −0.66]**	**0.0000**
MRI abnormal	**−2.31**	**0.53**	**[−3.35, −1.28]**	**0.0000**	−0.19	0.28	[−0.73, 0.36]	0.5018
MRI normal	**−1.11**	**0.28**	**[−1.66, −0.55]**	**0.0001**	**−0.95**	**0.15**	**[−1.24, −0.65]**	**0.0000**
LP abnormal	**−2.58**	**0.61**	**[−3.77, −1.39]**	**0.0000**	−0.33	0.33	[−0.98, 0.31]	0.3128
LP normal	**−1.13**	**0.27**	**[−1.67, −0.60]**	**0.0000**	**−0.87**	**0.15**	**[−1.16, −0.58]**	**0.0000**
Total NPI Score								
With catatonia	**−7.40**	**0.59**	**[−8.55, −6.25]**	**0.0000**	**−3.22**	**0.50**	**[−4.20, −2.23]**	**0.0000**
Without catatonia	**−4.05**	**0.97**	**[−5.95, −2.14]**	**0.0000**	**−2.68**	**0.83**	**[−4.31, −1.05]**	**0.0012**
With any neurodiagnostic abnormalities	**−9.55**	**0.83**	**[−11.17, −7.93]**	**0.0000**	**−2.03**	**0.73**	**[−3.46, −0.61]**	**0.0052**
Without any neurodiagnostic abnormalities	**−4.91**	**0.68**	**[−6.24, −3.58]**	**0.0000**	**−4.05**	**0.60**	**[−5.22, −2.88]**	**0.0000**
EEG abnormal	**−10.00**	**1.06**	**[−12.08, −7.92]**	**0.0000**	**−2.11**	**0.91**	**[−3.90, −0.31]**	**0.0213**
EEG normal	**−5.62**	**0.63**	**[−6.87, −4.38]**	**0.0000**	**−3.64**	**0.55**	**[−4.71, −2.57]**	**0.0000**
MRI abnormal	**−10.13**	**1.17**	**[−12.42, −7.83]**	**0.0000**	*−1.81*	*0.99*	*[−3.76, 0.13]*	*0.0678*
MRI normal	**−5.82**	**0.62**	**[−7.05, −4.60]**	**0.0000**	**−3.64**	**0.53**	**[−4.68, −2.60]**	**0.0000**
LP abnormal	**−9.42**	**1.41**	**[−12.17, −6.66]**	**0.0000**	−1.67	1.15	[−3.92, 0.59]	0.1470
LP normal	**−6.25**	**0.63**	**[−7.48, −5.02]**	**0.0000**	**−3.55**	**0.51**	**[−4.56, −2.54]**	**0.0000**

*P* < 0.1 (italic font); *P* < 0.05 (bold font).

In total, there were 12 individuals who did not respond to therapy. Amongst non-responders, 11/12 (92%) had no neurodiagnostic study abnormalities, 11/12 (92%) had no altered mental status, 11/12 (92%) had no developmental regression, and 10/12 (83%) had no catatonia, although all met criteria for possible DSRD [3]. In addition, 8/12 (67%) had neither altered mental status, developmental regression or catatonia. Finally, there were no statistical differences with regard to the likelihood of response (*P* = 0.69, 95% CI: 0.57–2.33) or degree of response on clinical assessments (*P* = 0.41, 95% CI: 0.63–1.56) between different formulations of IVIg.

### Neurodiagnostic abnormalities and disease severity

Patients with any neurodiagnostic abnormalities had higher means at baseline for 25FTW (MD: 3.50; 95% CI: 0.84, 6.15) and Total NPI (MD: 3.16; 95% CI: 0.42, 5.90) compared to those without any abnormalities. A lower mean of CGI-S (MD: −0.81; 95% CI: −1.26, −0.37) was observed for patients with any neurodiagnostic abnormalities while on therapy compared to baseline. Moreover, patients with any neurodiagnostic abnormalities had higher mean scores of 25FTW (MD: 4.67; 95% CI: 2.70, 7.07), BFCRS (MD: 6.69; 95% CI: 2.40, 10.98), CGI-S (MD:1.13; 95% CI: 0.43, 1.84), and Total NPI (MD: 5.17; 95% CI: 2.51, 7.83) after therapy compared to those without any abnormalities. A borderline difference was observed between patients with and without neurodiagnostic abnormalities while on therapy (MD: 1.35; 95% CI: −0.002, 2.71).

With regards to specific neurodiagnostics, patients with EEG abnormality had lower mean for BFCRS and NPITS while on therapy and after therapy relative to baseline. Similar pattern was observed for patients without EEG abnormality while on therapy and after therapy compared to baseline. Patients with abnormal EEG had lower mean NPITS while on therapy (MD: −3.54; 95% CI: −6.04, −1.05) compared to those without EEG abnormality. Patients with abnormal neuroimaging had lower means for BFCRS, CGI-S, and NPITS while on therapy compared to baseline. Similarly, patients without neuroimaging abnormalities had lower BFCRS, CGI-S, and NPITS while on therapy and also after therapy compared to baseline. While patients with abnormal neuroimaging had lower mean for CGI-S while on therapy (MD: −0.69; 95% CI: −1.24, −0.14) compared to those with normal neuroimaging, they had higher means of CGI-S (MD: 1.28; 95% CI: 0.44, 2.12) and NPITS (MD: 5.00; 95% CI: 1.76, 8.24) after therapy compared to patients with normal neuroimaging.

Patients with LP abnormalities had lower means of BFCRS, CGI-S, and NPITS while on therapy compared to baseline. Those with a normal LP had lower mean levels of BFCRS, CGI-S, and NPITS while on therapy and after therapy relative to baseline. Patients with abnormal LP had higher mean levels at baseline for CGI-S (MD: 0.93; 95% CI: 0.04, 1.83) and NPITS (MD: 3.88; 95% CI: 0.26, 7.51) compared with patients without such abnormality. Moreover, higher mean levels for CGI-S (MD: 1.47; 95% CI: 0.53, 2.40) and NPITS (MD: 5.77; 95% CI: 2.17, 9.36) were observed for patients with LP abnormality after therapy compared to those without abnormality.

### Change in clinical features incorporating neurologic relapse

**Fig. 2:**
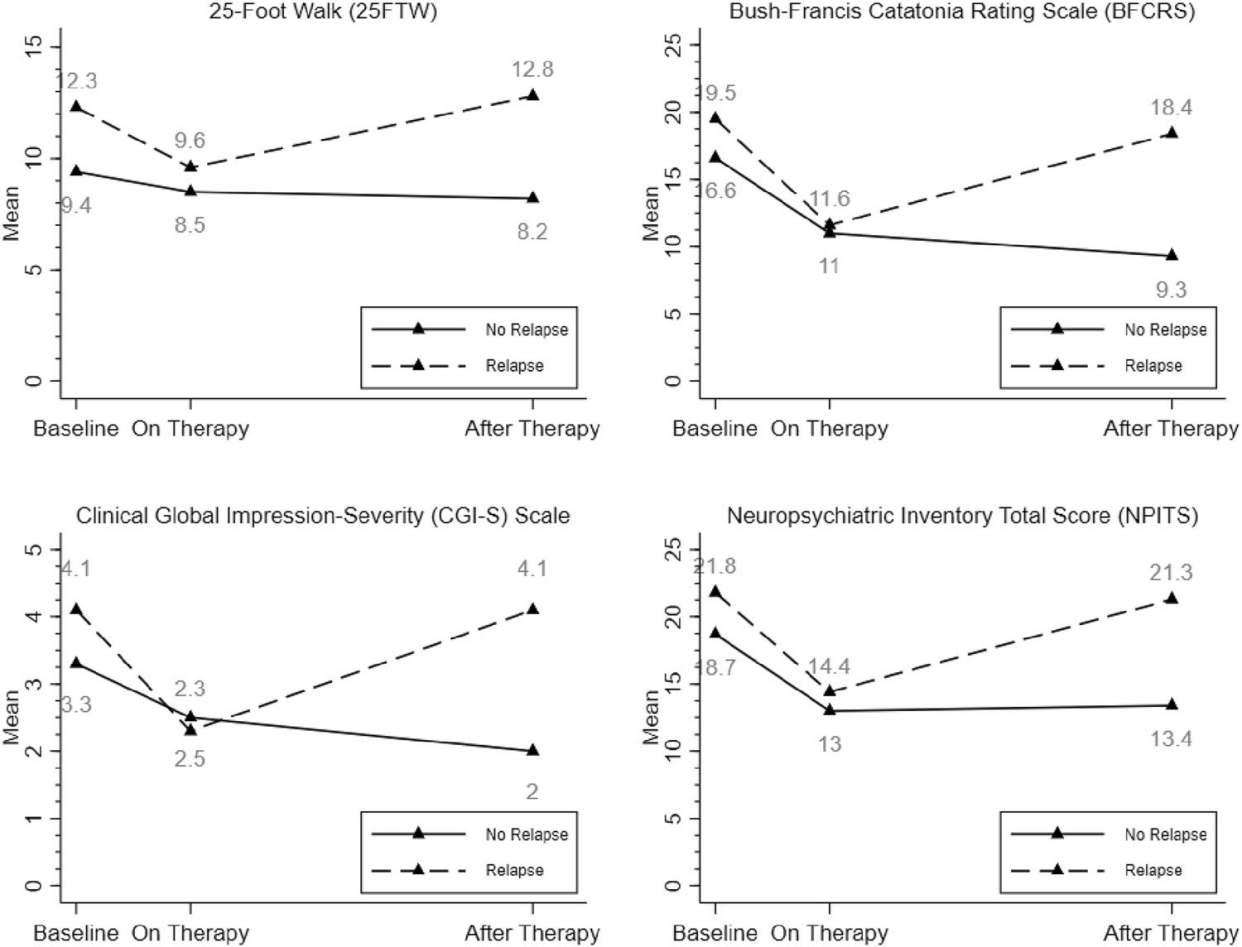
Longitudinal Therapeutic Responses in Individuals with and without Relapse. Clinical features, including behavioral and neuropsychiatric assessments over the study period for patients with and without neurologic relapse.

The therapeutic response across all clinical measures was sustained in individuals who did not relapse although those that did relapse had scores return to baseline levels (Fig. 2). We observed a significant reduction in mean scores of all clinical outcomes while on therapy compared to baseline for patients with relapse (Appendix C). For patients without relapse, there was also evidence of reduction in means of all the outcomes while on therapy compared to baseline, except for 25FTW. In addition, significant reduction in scores were observed among these patients without relapse when comparing after therapy with baseline. Patients with relapse had higher baseline means for all the clinical outcomes, except for BFCRS and higher mean scores for all the clinical outcomes after therapy compared to patients without relapse (Appendix D).

### Clinical features and relapse

There was evidence that the association between clinical responses and sex, catatonia, any neurodiagnostic abnormalities, and treatment with prior immunotherapy differed by neurologic relapse. Of note, individuals with any neurodiagnostic abnormality who did relapse had lower mean scores on the 25FTW while on therapy and higher mean after therapy relative to baseline. Sub-analysis of the impact of non-IVIg medications (e.g., anti-depressants) on relapse was not possible due to highly heterogenous treatments, yielding only 12 patients with identical regimens.

### Risk of relapse and neurodiagnostic abnormalities

**Fig. 3:**
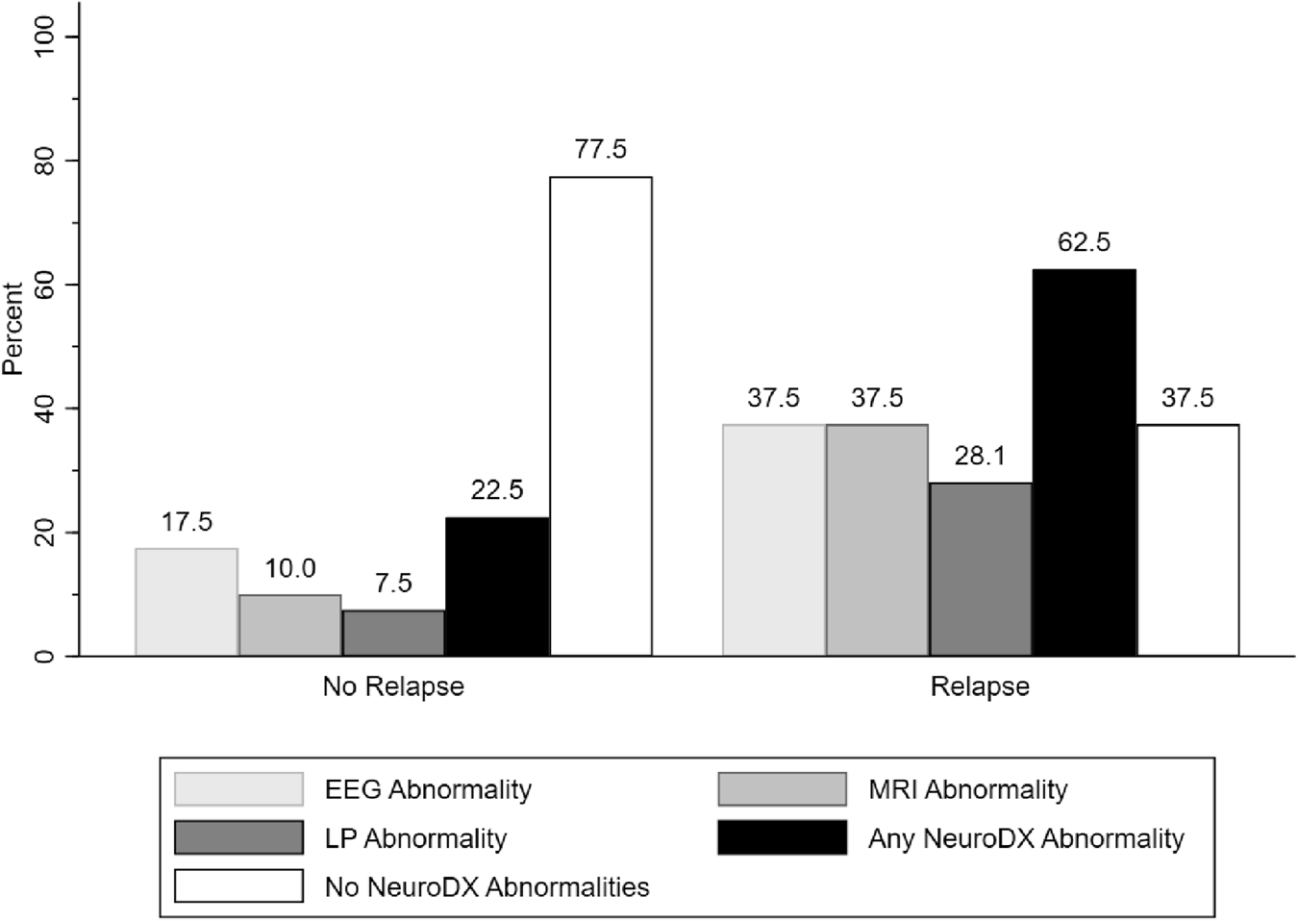
Prevalence of Neurodiagnostic Abnormalities in Individuals with and without Relapse. Neurodiagnostic abnormality presence in patients with (*n* = 44) and without (*n* = 38) neurologic relapse.

Approximately 46% of the patients (*n* = 38) experienced a relapse of symptoms. Patients who relapsed were more likely to have any abnormal neurodiagnostic study (*χ*^2^ = 11.82; *P* = 0.001), abnormal MRI (*χ*^2^ = 7.78; *P* = 0.005), and abnormal LP (*χ*^2^ = 5.45; *P* = 0.02) compared to patients without relapse (Fig. 3). Individuals with a history of personal autoimmunity were six times more likely to relapse than those without (OR: 6.11, *P* < 0.001, 95% CI: 2.69–12.13). In addition, patients with relapse had higher baseline 25FTW (*P* = 0.0222), CGI Severity of illness Score (*P* = 0.0152), NPI Irritability Score (*P* = 0.005), and NPITS (*P* = 0.0171) than those without relapse (Table 1).

### Predictors of relapse

#### Unadjusted analysis

There was no evidence of significant association between individual demographic characteristics and neurologic relapse after therapy, except a borderline association with the difference between age at therapy and age at diagnosis (OR: 2.20, 95% CI: 0.98, 4.93). Higher odds of relapse were associated with MRI abnormality (OR: 5.40, 95% CI: 1.54, 18.97), LP abnormality (OR: 4.83, 95% CI: 1.18, 19.70), and any neurodiagnostic abnormality (OR: 5.74, 95% CI: 2.05, 16.10).

Relapse was associated with a number of baseline clinical features, where the odds of relapse increased by one unit for each increase in 25FTW (OR: 1.11, 95% CI: 1.01, 1.21), CGI-S (OR: 1.49, 95% CI: 1.07, 2.07), NPI irritability (OR: 1.65, 95% CI: 1.14, 2.39), and NPITS (OR: 1.10, 95% CI: 1.01, 1.19). In addition, borderline associations were observed between relapse and baseline NPI Hallucinations (OR: 1.31, 95% CI: 0.97, 1.77) and NPI Apathy (OR: 1.32, 95% CI: 0.98, 1.77) scores.

#### Adjusted analysis

The disease biometrics and baseline clinical features that differed significantly by relapse were included in a model adjusting for sex, ethnicity, and difference between age at therapy and age at diagnosis. Analysis revealed a greater risk of relapse after therapy was associated with any neurodiagnostic abnormality (aOR: 4.34, 95% CI: 1.39, 13.53). No evidence of significant association was observed between relapse and other baseline covariates was present.

## Discussion

Individuals with DSRD were responsive to immunotherapy on a variety of clinical measures, consistent with previously published data [4, 9, 10]. Patients demonstrated improvements in functional status (CGI), gait (25FTW), catatonia (BFCRS and 25FTW), and neuropsychiatric symptoms (NPITS). Further, treatment of these patients for a period of 9–12 months yielded sustained improvement in 47% of individuals after IVIg immunotherapy was weaned off. Those with a history of personal autoimmunity or baseline neurodiagnostic abnormalities were more likely to experience a clinical relapse upon wean of immunotherapy. These findings advance our understanding of the DSRD phenotype having immunologic origins in a subset of individuals.

Beneficial clinical responses to immunotherapy (IVIg) were high in this cohort at roughly 85% (70/82), consistent with prior studies [4, 9, 10]. These improvements were most notable in individuals with catatonia or neurodiagnostic study abnormalities, which is consistent with previously published data [4]. Non-responders to therapy were less likely to have neurodiagnostic study abnormalities (7%), altered mental status (7%), developmental regression (7%), and catatonia (17%). Although these findings were observed in a limited cohort (*n* = 12), it does indicate that some clinical features could be more predictive of non-immunotherapy-responsive disease. Conversely, this study also expands on the concept that even in patients without definitive neurodiagnostic abnormalities, there may be a role for immune-based interventions as well, highlighting need to prioritize identification of sensitive and specific biomarkers in DSRD.

In addition to the clinical responses observed in this study, the safety of the administration of IVIg should be noted. Only 2.4% of patients (*n* = 2) had any adverse event during the administration period, and these were mild, self-limited, and did not recur. Importantly, no participant developed deep venous thrombosis or clotting, headache, aseptic meningitis, or other known side effects of IVIg. Although further assessment of risk will be needed in real-world cohorts, these are particularly encouraging data given concerns of the tolerability of IV infusions and IVIg in this population.

Duration of immunotherapy has remained an important question in individuals with DSRD since the first cohorts of immunotherapy-responsive patients were published [10]. This study used a slow therapeutic wean, consistent with previously published literature, in order to avoid rebound after abrupt discontinuation [18, 19]. Ultimately, the 53% of individuals that did relapse upon wean of IVIg may represent a cohort of individuals where the etiology of their DSRD-related symptoms is potentially inflammatory in nature. Etiologies to DSRD remain poorly elucidated although emerging evidence for a neuroinflammatory component to the disease in a minority of individuals has gained traction [3, 9]. Thus, the data presented in this report not only serves to highlight the therapeutic effect of IVIg in DSRD, but also provides proof of concept that the immunomodulatory effects of this therapeutic may be treating a potential, albeit unknown, inflammatory target. This is supported the observed 4.34 times greater risk of relapse in individuals with neurodiagnostic study abnormalities (including early/accelerated mineralization and CSF abnormalities) which are indicative of potentially interferon-driven immune dysregulation, a concept more well-established in systemic disease presentations in persons with DS [20–23]. It has been established that individuals with DS are at great risk for a variety of autoimmune disorders [24–30], and thus it would be reasonable to consider the brain as another potential target of immune dysregulation.

In this study, relapse was treated with a re-initiation of the previously employed IVIg protocol of administration of 1 g/kg every 4 weeks. While this intervention did stabilize patients, the uncertainty of duration of therapy in patients who experience relapse off of IVIg remains. At this time, there are no longitudinal data to support indefinite IVIg although prior reports have demonstrated success with a variety of second-line immunotherapies including mycophenolate mofetil, azathioprine, and rituximab [4]. The authors support consideration of these therapeutics when there is evidence of neurodiagnostic study abnormalities although individuals without these findings often lack profound responses to these treatments [4] and should be evaluated on a case-by-case basis given the limited data available at this time.

This study is not without limitations. Selection and severity bias is present in the exclusion of patients who did not undergo neurodiagnostic workup. Investigators involved in this study were early adopters of immunotherapy in persons with DSRD, and thus both a referral and selection bias in favor of use of IVIg may be present. Severity bias could also decrease the likelihood of response to any therapy, but the authors do contemplate if more severe cases are associated with inflammatory etiologies which is still undetermined. This same severity bias could have explained the responsiveness to IVIg and the high rates of neurologic relapse in this data set. The authors also included four patients who had previously received incomplete immunotherapy regimens over 6 months prior to re-starting IVIg which could have influenced the likelihood of response to immunotherapy. Sub-analysis of non-immunotherapy was not possible in this study due to the low number of patients on the same regimen. Clinical treatment heterogeneity, utilization of weight-based dosing and lack of algorithmic therapeutic interventions in DSRD are major limiting factors in this disease and yielded only 12 patients who were on exactly the same treatments at the time of immunotherapy initiation. Future studies, including an upcoming clinical trial set to launch in late 2023, will focus on the prospective collection of the efficacy of monotherapies in the treatment of DSRD which will help elucidate the efficacy of each individual therapeutic in this condition. Steroids were administered on six occasions as reaction medications during this study and could have potentially influenced the rate of response as steroids have been previously shown to treat DSRD in a minority of patients [4]. However, the very low number of administrations (0.5% of all infusions during the treatment period) make this unlikely. In addition, patients were on a variety of different psychotropic medications at baseline and while these were not changed during this study, add an additional layer of complexity to interpreting and generalizing the results. With regards to metrics, the use of CGI is subjective measure prone to recall bias, although this was mitigated by the use of two objective, physician-based metrics. This study did not assess family response to treatments (e.g., functional improvements in homelife for family), although the authors acknowledge this would be an important variable to assess in future study. Importantly, this study was not randomized nor controlled which should temper interpretation. Standardized immunotherapy regimens and wean schedules mitigated some of this although further randomized, controlled, trials are desperately needed in this space. Different formulations of IVIg had to be used although no significant differences between formulations were identified. In addition, in our demographic and clinic response variable analysis, we observed statistical (and lack thereof) differences in study tools; this may reflect differences in disease severity between those comparison groups or more likely reflect the study’s low power. Finally, the authors note that given the rare nature of DSRD, low study populations limit the generalizability of these findings and the authors caution clinicians to evaluate each case individually.

In summary, amongst a cohort of individuals with DSRD, immunotherapy was safe and effective in the treatment of the clinical symptom cluster. Individuals with neurodiagnostic abnormalities of any type were significantly less likely to be able to wean off of immunotherapy, indicating the potential for, a chronic immune etiology in some cases of DSRD. These results must be tempered by multiple study limitations, although provide a basis for further investigations into randomized controlled, double-blinded, biomarker and therapeutic trials in this emerging disease.

### Supplementary information

Appendix A

Appendix B

Appendix C

Appendix D
