# Supplementary material for: Immunotherapy responsiveness and risk of relapse in Down syndrome regression disorder
Source: Transl Psychiatry. 2023 Aug 8;13:276. doi: 10.1038/s41398-023-02579-z (PMC10409776; doi:10.1038/s41398-023-02579-z)
Supplement: Supplementary file 1 — Appendix A [file 41398_2023_2579_MOESM1_ESM.pdf]

Appendix A: Protocol Graphical Timeline

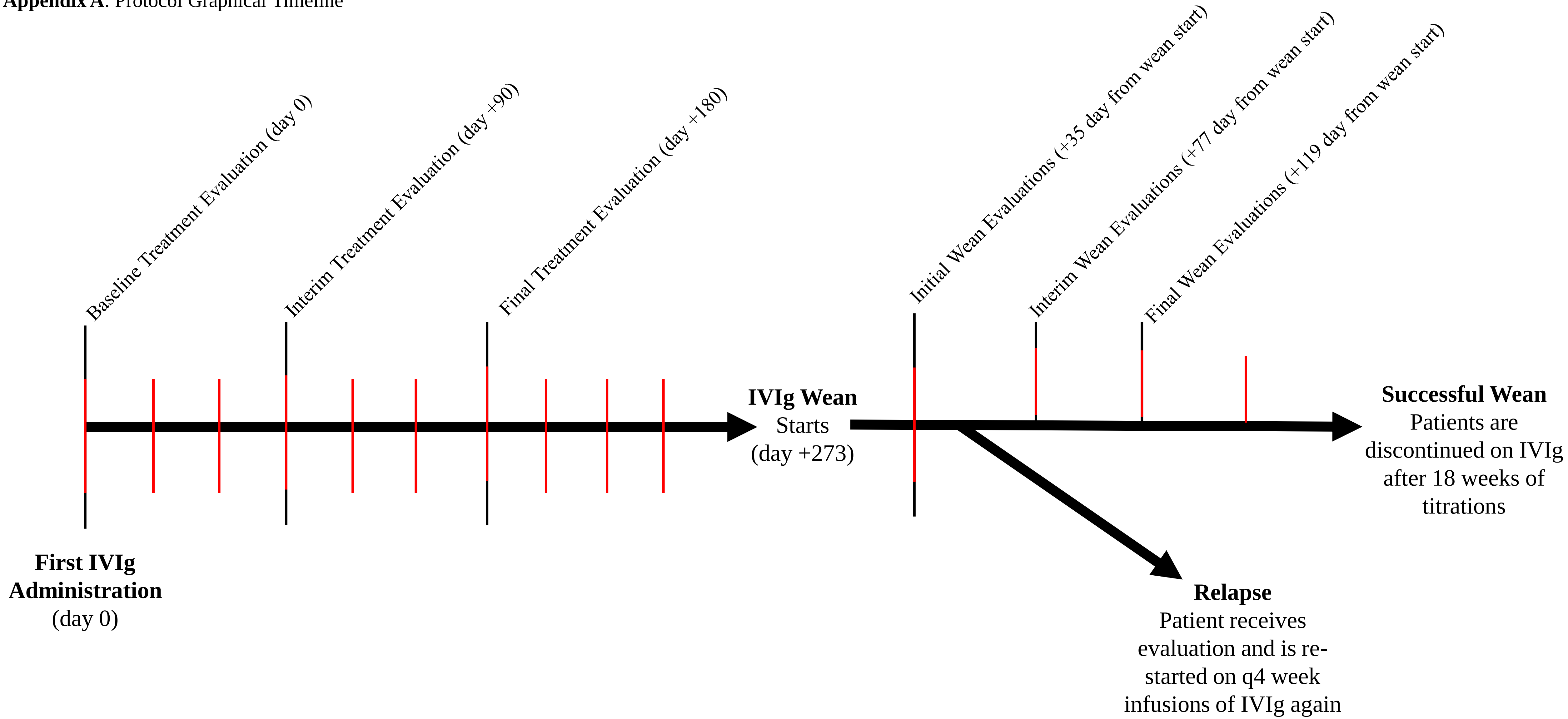

Legend: *black bars indicate evaluation; red bars indicate administration of IVIg. Initial administration of IVIg is 2 g/kg over two days and subsequent dosing is 1 g/kg thereafter. Relapse could occur at any time point after wean was initiated although is represented after the first titration which was the common time point for clarity purposes.*
