# Supplementary material for: Immunotherapy responsiveness and risk of relapse in Down syndrome regression disorder
Source: Transl Psychiatry. 2023 Aug 8;13:276. doi: 10.1038/s41398-023-02579-z (PMC10409776; doi:10.1038/s41398-023-02579-z)
Supplement: Supplementary file 2 — Appendix B [file 41398_2023_2579_MOESM2_ESM.docx]

**Appendix B:** Infusion Protocol

1. Monitoring
   1. Notify MD if [all patients]
      1. Temperature > 38.0 C or <36.0 C
      2. Blood pressure [per age range]
      3. Respiratory Rate [per age range]
   2. Obtain Height and Weight [all patients]
   3. Cardiorespiratory monitoring [as clinically indicated]
   4. Monitor and record presence/absence of infusion related reactions: fever, chills, pruritus, urticaria, chest pain, hypertension, or dyspnea. Notify MD if infusion reaction occurs & stop infusion immediately
   5. Pulse Oximetry [as clinically indicated]
2. Pre-Infusion
   1. Acetaminophen
      1. 10 mg/kg, max 650 mg PO
   2. Diphenhydramine
      1. 1 mg/kg, max 50 mg PO
      2. OR 1 mg/kg, max 50 mg IV [if medically necessary]
   3. Methylprednisolone
      1. 1 mg/kg or max 60 mg IV [if clinically indicated]
   4. Omeprazole
      1. 1 mg/kg or max 20 mg PO [if clinically indicated]
3. Emergency Medications – only administered if concern for infusion reaction
   1. Epinephrine (1 mg/mL = 1:1000]
      1. 0.3 (pediatric) or 0.5 mg (adult) IM
   2. Methylprednisolone
      1. 1 mg/kg or 60 mg IV
   3. Diphenhydramine
      1. 1 mg/kg or 50 mg IV
   4. If sustained symptoms after dosing, patient must be transported to local ED
4. PRN Medications
   1. Diphenhydramine
      1. 1 mg/kg or 50 mg PO q6h prn rash or vomiting
   2. Acetaminophen
      1. 10 mg/kg or 650 mg PO q4h prn rash, pain or headache
   3. Furosemide
      1. 0.5 mg/kg IV x1 for fluid overload
   4. Albuterol (0.083% inhaled solution)
      1. 2.5 mg nebulized prn wheezing/respiratory distress
   5. Sodium chloride (0.9% NS)
      1. 20 mL/kg or max 1000 mL prn hypotension or tachycardia
5. Medication Dosing
   1. All dosing regimens
      1. First dose: 2 g/kg divided over two consecutive days
      2. Maintenance doses: 1 g/kg over one day, administered every four weeks
   2. Immune Globulin (Gammagard 10%)
      1. Concentration: 10%
      2. pH: 6.4-7.2
      3. Initial Rate: 0.5 mL/kg/hr
      4. Max Rate: 8 mL/kg/hr
      5. IgA Content: <2 mcg/mL
      6. Osmolarity: 1250 mOsmol/kg
      7. Note: contains Polysorbate 80
   3. Immune Globulin (Octagam 10%)
      1. Concentration: 10%
      2. pH: 4-4.5
      3. Initial Rate: 0.6 mL/kg/h
      4. Max Rate: 4.8 mL/kg/hr
      5. IgA Content: 46 mcg/mL
      6. Osmolarity: 258 mOsmol/kg
   4. Immune Globulin (Privagen 10%)
      1. If clinically indicated, patient must have impaired renal clearance or require sugar-free formulation
      2. Concentration: 10%
      3. pH: 4.6-5.0
      4. Initial Rate: 0.3 mL/kg/hr
      5. Max Rate: 4.8 mL/kg/hr
      6. IgA Content: <25 mcg/mL
      7. Osmolarity: 240-440 mOsmol/kg
      8. Note: Contains L-proline
