# Supplementary material for: Immunotherapy responsiveness and risk of relapse in Down syndrome regression disorder
Source: Transl Psychiatry. 2023 Aug 8;13:276. doi: 10.1038/s41398-023-02579-z (PMC10409776; doi:10.1038/s41398-023-02579-z)
Supplement: Supplementary file 3 — Appendix C [file 41398_2023_2579_MOESM3_ESM.docx]

| **Appendix C**: Estimated means with 95% confidence interval [CI] for the outcomes of interest by relapse | | | | | | | | | |
| --- | --- | --- | --- | --- | --- | --- | --- | --- | --- |
|  | On-Therapy | | | |  | After-Therapy | | | |
|  | Mean change from baseline | SE | 95% CI | p-value |  | Mean change from baseline | SE | 95% CI | p-value |
| *25-Foot walk* |  |  |  |  |  |  |  |  |  |
| Relapse | **-2.69** | **0.51** | **[-3.69, -1.70]** | **0.0000** |  | 0.57 | 0.41 | [-0.23, 1.38] | 0.1622 |
| No Relapse | *-0.87* | *0.47* | *[-1.80, 0.05]* | *0.0650* |  | **-1.12** | **0.38** | **[-1.87, -0.38]** | **0.0032** |
|  |  |  |  |  |  |  |  |  |  |
| *Bush-Francis Score* |  |  |  |  |  |  |  |  |  |
| Relapse | **-7.89** | **1.15** | **[-10.15, -5.64]** | **0.0000** |  | -1.08 | (0.98) | [-2.99, 0.84] | 0.2693 |
| No Relapse | **-5.64** | **1.07** | **[-7.73, -3.54]** | **0.0000** |  | **-7.32** | **0.91** | **[-9.10, -5.54]** | **0.0000** |
|  |  |  |  |  |  |  |  |  |  |
| *CGI-Severity Score* |  |  |  |  |  |  |  |  |  |
| Relapse | **-1.79** | **0.34** | **[-2.45, -1.13]** | **0.0000** |  | 0.00 | 0.15 | [-0.29, 0.29] | 1.0000 |
| No Relapse | **-0.82** | **0.31** | **[-1.43, -0.21]** | **0.0088** |  | **-1.32** | **0.14** | **[-1.59, -1.05]** | **0.0000** |
|  |  |  |  |  |  |  |  |  |  |
| *Total NPI Score* |  |  |  |  |  |  |  |  |  |
| Relapse | **-7.45** | **0.77** | **[-8.95, -5.95]** | **0.0000** |  | -0.55 | 0.50 | [-1.54, 0.43] | 0.2721 |
| No Relapse | **-5.68** | **0.71** | **[-7.08, -4.29]** | **0.0000** |  | **-5.25** | **0.47** | **[-6.17, -4.33]** | **0.0000** |

^*^*^p^* ^< 0.1 (italic font); **^*^p^* ^< 0.05 (bold font).^
