# Supplementary material for: Immunotherapy responsiveness and risk of relapse in Down syndrome regression disorder
Source: Transl Psychiatry. 2023 Aug 8;13:276. doi: 10.1038/s41398-023-02579-z (PMC10409776; doi:10.1038/s41398-023-02579-z)
Supplement: Supplementary file 4 — Appendix D [file 41398_2023_2579_MOESM4_ESM.docx]

| **Appendix D**: Estimated means with 95% confidence interval [CI] for the outcomes of interest by time | | | | |
| --- | --- | --- | --- | --- |
|  | Relapse | | | |
|  | Mean change compared to patients without relapse | SE | 95% CI | p-value |
| 25-Foot walk |  |  |  |  |
| Prior to Therapy^**^ | **2.91** | **1.21** | **[0.54, 5.28]** | **0.0163** |
| On-Therapy^*^ | *1.08* | *0.62* | *[-0.13, 2.30]* | *0.0809* |
| After-Therapy^**^ | **4.60** | **1.06** | **[2.51, 6.69]** | **0.0000** |
|  |  |  |  |  |
| Bush-Francis Score |  |  |  |  |
| Prior to Therapy | 2.91 | 2.29 | [-1.59, 7.41] | 0.2049 |
| On-Therapy | 0.65 | 1.63 | [-2.54, 3.84] | 0.6896 |
| After-Therapy^**^ | **9.15** | **1.87** | **[5.48, 12.81]** | **0.0000** |
|  |  |  |  |  |
| CGI-Severity Score |  |  |  |  |
| Prior to Therapy | **0.78** | **0.31** | **[0.17, 1.39]** | **0.0118** |
| On-Therapy^**^ | -0.19 | 0.22 | [-0.63, 0.24] | 0.3883 |
| After-Therapy^**^ | **2.10** | **0.25** | **[1.61, 2.58]** | **0.0000** |
|  |  |  |  |  |
| Total NPI Score |  |  |  |  |
| Prior to Therapy^**^ | **3.13** | **1.28** | **[0.62, 5.65]** | **0.0145** |
| On-Therapy | 1.37 | 1.08 | [-0.76, 3.49] | 0.2071 |
| After-Therapy^**^ | **7.83** | **1.06** | **[5.76, 9.91]** | **0.0000** |

^*^*^p^* ^< 0.1 (italic font); **^*^p^* ^< 0.05 (bold font).^
